# Spectrum of MODY in the south of New Zealand – including two novel mutations

**DOI:** 10.1186/1687-9856-2013-S1-P39

**Published:** 2013-10-03

**Authors:** N Patterson, B Taylor, G Dainty, D Prosser, P Tomlinson, DR Love, B Wheeler

**Affiliations:** 1 Southern District Health Board, Otago/Southland, New Zealand; 2 University of Otago, Dunedin, New Zealand; 3 Diagnostic Genetics, LabPlus, Auckland, New Zealand; 4 School of Biological Sciences, University of Auckland, Auckland, New Zealand; 5 Edgar National Centre for Diabetes and Obesity Research, Dunedin, New Zealand

## 

Maturity Onset Diabetes of the Young (MODY) is a monogenic form of diabetes. It consists of a heterogeneous group of autosomal dominant inherited disorders, with typical onset in individuals aged less than twenty five years. There are several different sub-types of MODY which can be precisely identified using molecular genetic testing. Modes of presentation vary and can mimic type 1 or 2 diabetes. Making a specific diagnosis of MODY can have important implications for guidance of appropriate treatment, prognosis and genetic counselling.

The paediatric diabetes team across the Southern District Health Board provides diabetes care to approximately 160 children and adolescents spread over the largest geographical region in New Zealand. We present the cases of three children and their families diagnosed with MODY over the past two years. This includes two novel mutations, one of which segregates in a unique kindred that is strongly affected by both MODY and classic autoimmune mediated diabetes.

These families highlight the features of three of the more common MODY subtypes; MODY2 - a 13 year old boy with a novel unclassified variant c.698G>A (p.Cys233Tyr) in exon 7 of the Glucokinase (GCK) gene, MODY5 – a 14 year old girl with a novel de novo dosage change of a 1.3MB deletion in the HNF1β gene, and MODY3 with a reported nonsense mutation c.864delGinsCC (p.Gly282ArgfsX25) in exon 4 of the HNF1α gene observed in an 11 year old girl. In addition to these index cases, genetic testing has lead to the diabetes diagnosis of a sibling with a GCK mutation, and the identification of an HNF1α mutation in another currently asymptomatic sibling. To date, we have identified a prevalence of MODY in the paediatric diabetes population of the lower South Island of approximately 2.5%. This prevalence, along with increasing access to molecular genetic testing, highlights the importance of consideration of MODY in atypical diabetes presentations in the paediatric/adolescent population.

